# Supplementary material for: Prognostic factors and quality of life after pelvic fractures. The Brabant Injury Outcome Surveillance (BIOS) study
Source: PLoS One. 2020 Jun 11;15(6):e0233690. doi: 10.1371/journal.pone.0233690 (PMC7289384; doi:10.1371/journal.pone.0233690)
Supplement: S1 Protocol — (PDF) [file pone.0233690.s001.pdf]

Downloaded from http://injuryprevention.bmj.com/ on May 8, 2016 - Published by group.bmj.com
IP Online First, published on May 6, 2016 as 10.1136/injuryprev-2016-042032

Prevalence, recovery patterns and predictors of quality of life and costs after non-fatal injury: the Brabant Injury Outcome Surveillance (BIOS) study

M A C de Jongh,[1,2] N Kruithof,[1] T Gosens,[1,3] C L P van de Ree,[1] L de Munter,[1] L Brouwers,[2] S Polinder,[4] K W W Lansink,[1,2,5] BIOS-group

[1]Department Trauma TopCare, Elisabeth-TweeSteden Hospital, Tilburg, The Netherlands
[2]Network Emergency Care Brabant, Brabant Trauma Registry, The Netherlands
[3]Department of Orthopaedics, Elisabeth-TweeSteden Hospital, Tilburg, The Netherlands
[4]Department of Public Health, Erasmus MC, Rotterdam, The Netherlands
[5]Department of Surgery, Elisabeth-TweeSteden Hospital, Tilburg, The Netherlands

Correspondence to
Dr M A C de Jongh, Department of Trauma TopCare, Elisabeth-TweeSteden Hospital, Postbus 90151, Tilburg 5000LC, The Netherlands; m.dejongh@etz.nl

Received 13 March 2016
Revised 15 March 2016
Accepted 2 April 2016

To cite: de Jongh MAC, Kruithof N, Gosens T, *et al*. *Inj Prev* Published Online First: [*please include* Day Month Year] doi:10.1136/injuryprev-2016-042032

ABSTRACT

Introduction Trauma is a major public health problem worldwide that leads to high medical and societal costs. Overall, improved understanding of the full spectrum of the societal impact and burden of injury is needed. The main purpose of the Brabant Injury Outcome Surveillance (BIOS) study is to provide insight into prevalence, predictors and recovery patterns of short-term and long-term health-related quality of life (HRQoL) and costs after injury.

Materials and methods This is a prospective, observational, follow-up cohort study in which HRQoL, psychological, social and functional outcome, and costs after trauma will be assessed during 24 months follow-up within injured patients admitted in 1 of 10 hospitals in the county Noord-Brabant, the Netherlands. Data will be collected by self-reported questionnaires at 1 week (including preinjury assessment), and 1, 3, 6, 12 and 24 months after injury. If patients are not capable of filling out the questionnaires, proxies will be asked to participate. Also, information about mechanism and severity of injury, comorbidity and indirect and direct costs will be collected. Mixed models will be used to examine the course of HRQoL, functional and psychological outcome, costs over time and between different groups, and to identify predictors for poor or good outcome.

Relevance This study should make a substantial contribution to the international collaborative effort to assess the societal impact and burden of injuries more accurately. The BIOS results will also be used to develop an outcome prediction model for outcome evaluation including, besides the classic fatal, non-fatal outcome.

Trial registration number NCT02508675.

INTRODUCTION

Trauma is a major public health problem worldwide that remains one of the leading causes of death and disability and also leads to high medical and societal costs.[1 2]

Over the past decades, case fatality rates of severe injury have rapidly decreased, especially in countries with advanced health systems.[3] This puts a growing number of patients at risk of serious long-term disability.[4 5] In other words, the burden of trauma has shifted largely from fatal to non-fatal outcome. Many of these patients with non-fatal injury are young people, whose daily activities like work and leisure may suffer greatly after trauma.

Improved understanding of the consequences of non-fatal injuries is needed for the evaluation of treatment approaches, to be able to guide policymakers in prioritising of injury prevention research, to facilitate the (economic) evaluation of interventions and to contribute to international efforts to more accurately assess the burden of non-fatal injuries. Although trauma is recognised as a leading cause of morbidity, there is worldwide a shortage of systematic and population-based injury follow-up data collection to inform understanding of the predictors and the multidimensional consequences of non-fatal injury.[6 7] Integrated knowledge of medical, physical, psychological, societal consequences and costs of injuries is scarce.

There is need for an improved understanding of injury outcomes, better identification of risk groups of poor outcomes and new insights into how disability following injury can be reduced.[6] Up till now, there is insufficient systematic and population-based data collection and linkage to hospital data registries and trauma registries to fill this knowledge gap. Several prospective follow-up studies measuring the outcomes after trauma for a general injury population have been conducted nationwide and worldwide.[8–15] However, only a few studies covered the wide range of outcomes. Traditionally, burden-of-injury studies have focused on a single outcome measure, for example, the physical consequences of injury, health-related quality of life (HRQoL) or return to work. Furthermore, only a few follow-up studies extend beyond 1 year after trauma,[16] although residual disability at 1 year is often assumed to be perpetual. Besides this, most studies have been limited by small study size and substantial loss to follow-up.

Sound follow-up data on the incidence, severity and duration of the functional consequences and medical and societal costs of non-fatal injuries are needed. Data on all dimensions of functioning relevant to non-fatal injuries are needed to describe the pattern and risk factors of short-term and long-term outcome of injury patients over time. With the help of these data, the impact of injury on population health over time can be quantified.

Measuring the impact of injury is particularly challenging due to the large variation in injury types and severity. Therefore, it is important that valid methods will be used to estimate non-fatal injury outcome.

An important aspect is the choice of the study population. Although the association between severity of injury and long-term outcome is unclear,[17] several studies included only specific injuries[18–20] or severely injured patients.[4 21–24] The

Copyright Article author (or their employer) 2016. Produced by BMJ Publishing Group Ltd under licence.

Downloaded from http://injuryprevention.bmj.com/ on May 8, 2016 - Published by group.bmj.com

definition of severe injury in these studies is mostly based on scores like the AIS and the ISS, which are correlated to survival chances and not to permanent disability after injury. To give a complete insight into the risk factors and recovery patterns of non-fatal injuries, a broad inclusion of injuries and severity levels is necessary.

Furthermore, it is important to measure a wide range of outcomes. Only a few follow-up studies measured psychological consequences such as post-traumatic stress disorder (PTSD) and depression. However, numerous studies have shown that psychological problems occur relatively frequently among trauma patients.[17 25–28]

Furthermore, comprehensive and detailed information on direct healthcare costs and productivity costs will help to identify injuries and high-risk groups. A small number of studies described the medical and societal costs (eg, productivity loss) after injuries. However, costs enable rapid comparisons among very different types of injury. Intramural, extramural and societal costs can be high within the whole spectrum of injury patients.

In a former study, injury type, age, gender, length of hospital stay, intensive care unit (ICU) days, injury severity, post-traumatic stress symptoms and return to work were found to be associated with functional outcome and recovery.[25 26] Furthermore, important determinants of long-term disability after trauma are patients with one or more comorbidities,[29] patients with multiple injuries[30] and frailty in elderly patients.[31 32]

Besides these known risk factors, we will also focus on social economic status and job-related factors. In earlier follow-up studies, the importance of (a combination of) these determinants remained often understudied. Most studies do not include all these risk factors simultaneously, which restricts the possibility to adjust for confounding accurately. However, measuring and investigating risk factors besides outcome offers the opportunity to develop a prediction model and risk profiles for non-fatal outcome.

A large part of the non-fatal injury patients are elderly. According to recent literature, frailty places a patient at risk for a poor outcome following even a minor illness or injury and it is predictive for patients' mortality, postoperative complications and discharge to skilled nursing facilities.[31 33] Besides that, a frail patient is vulnerable to develop geriatric syndromes and to experience functional decline already during hospitalisation.[32]

Overall, improved understanding of the full spectrum of outcomes after injury is needed to better evaluate the predictors and recovery patterns after injury and to inform policymakers and guidelines to improve trauma care. Therefore, a population-based longitudinal survey of injured patients among the full spectrum of severity, including a large range of predictors and focusing on the multidimensional outcome after injury, is needed. This multidimensional approach is also needed to evaluate and improve the quality of trauma care.

Most outcome and performance evaluations of trauma care are classically based on mortality. However, the largest part of the trauma population survives. In the Netherlands, the mortality rate of the general acute hospitalised trauma population is 2%.[34] Moreover, the prevalence of decreased functioning will be higher than the mortality rate.

Many different risk-adjusted models were developed in the past decades to predict mortality in trauma patients.[35–38] A frequently used and cited model is the Trauma and Injury Severity Score (TRISS).[39] The TRISS is a logistic regression model of survival probability based on variables such as age, Revised Trauma Score [40] and ISS.[41 42] This model has been used in several countries.

In patients with traumatic brain injury (TBI), outcome models based on functional outcome and HRQoL have been established.[36 43 44] As far as we know, models for non-fatal outcome on different aspects for a complete clinical trauma population have never been developed. Therefore, our study aims to develop a valid, reliable and accurate prediction model for developing risk profiles for non-fatal outcome after injury.

This paper describes the protocol of the Brabant Injury Outcome Surveillance (BIOS) study. The BIOS is a prospective longitudinal follow-up study among all admitted injury patients in the region Noord-Brabant independent of severity or classification of injury to evaluate the total non-fatal burden of injury from a patient and societal perspective.

The overarching purpose of the project is to provide a multidimensional overview of short-term and long-term prevalence of morbidity and recovery patterns after injury. Furthermore, this will result in improving and developing risk profiles in the trauma population. It will also create a base for measuring, comparing and improving quality of trauma care using non-fatal outcome.

OBJECTIVES

1. to investigate the short-term and long-term HRQoL, functional, psychological and economic outcome after non-fatal trauma;
2. to investigate the risk factors for decreased HRQoL, functional, psychological and economic outcome after non-fatal trauma;
3. to describe the healthcare use, medical costs and productivity loss due to non-fatal trauma;
4. to develop a risk profile for recovery after non-fatal injury in the short and long term;
5. to validate and develop models for predicting non-fatal outcome after trauma;
6. to investigate whether a structural enlargement of the trauma registry with patient-reported outcome measurement does add value.

MATERIALS AND METHODS

Study design

The Brabant Trauma Registry (BTR) compiles prehospital and hospital data of all unintentional and intentional trauma patients admitted after presentation to the emergency department (ED) in 1 of 10 hospitals in the region Noord-Brabant (the Netherlands). The Dutch southern region Noord-Brabant has 2.4 million inhabitants, and about 12 000 injured patients are admitted annually. The BTR includes 10 hospitals, 12 EDs and 1 level 1 trauma centre. It covers representative amounts of urban and rural populations. As a result, the recorded injury incidence in the BTR is regarded as representative for the total population.

This is a prospective, observational, follow-up cohort study in which HRQoL, psychological and functional outcome, and costs after trauma will be assessed during 24 months follow-up within injured patients admitted in 1 of the 10 hospitals of the BTR. The inclusion period will be 1 year; from 1 August 2015 until 31 July 2016. A flow diagram of the project is shown in figure 1.

Participants

Adult injury patients who are seen at the ED, who will be admitted to an ICU or a ward in Noord-Brabant and who survived to hospital discharge will be included in the study. Both intentional and unintentional injuries and all types and severity of injury

Downloaded from http://injuryprevention.bmj.com/ on May 8, 2016 - Published by group.bmj.com

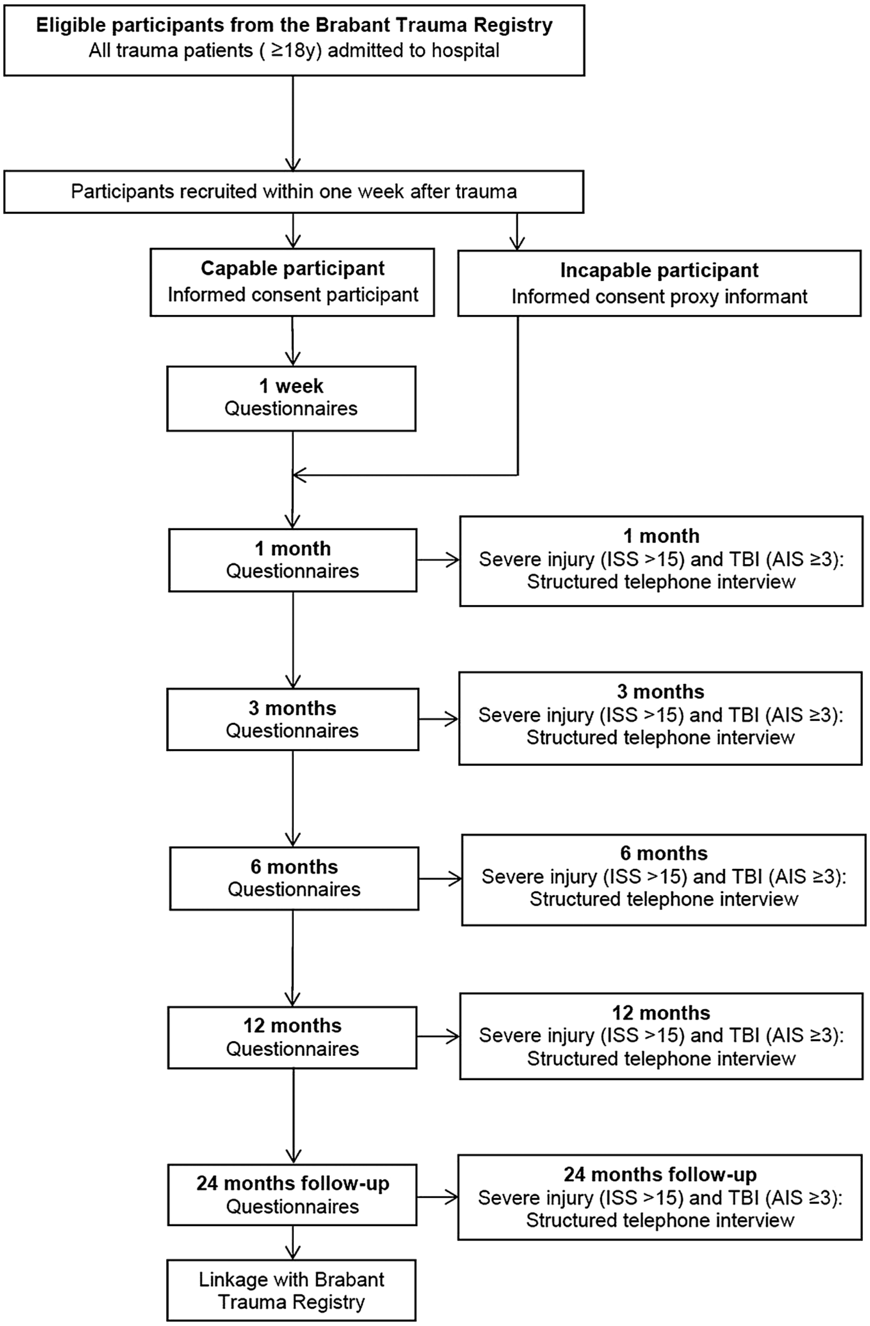

Figure 1 Flow diagram of the Brabant Injury Outcome Surveillance (BIOS) study. Abbreviations; ISS: Injury Severity Score; TBI: traumatic brain injury; AIS: Abbreviated Injury Scale.

will be included. A minimum age of 18 years and sufficient knowledge of the Dutch language are required. Patients with a pathological fracture caused by a malignancy or metastasis will be excluded.

In the region Noord-Brabant, there is no centre for the treatment of patients with severe burns. For that reason, patients with severe burns who are seen at the ED of a hospital in Noord-Brabant and who will be transferred to the nearest centre for patients with burns will be included as well.

If patients are incapable of completing the self-report measures themselves because of mental retardation, dementia or other neurological conditions, questionnaires will be completed by a proxy informant.

Data collection: registry data

Prehospital data (eg, vital signs and transport modes), type of injury, diagnosis, injury severity and in-hospital medical procedures will be obtained directly from the BTR to provide a comprehensive description of the population.

Socio-demographic characteristics

Patient characteristics (eg, age, sex, comorbidity, social economic status) will be electronically extracted from the BTR, Dutch Medical Registration and Electronic Medical Records and from the socio-demographic questions in the questionnaire.

Injury characteristics

Injury and admission data will be extracted from the BTR. The AIS (AIS-90, update 2008)[45 46] is used to define the anatomical region and severity of separate injuries in detail and can be used to determine multiple injury. The ISS[41] is used to assess overall trauma severity. To compute the ISS, each of the six anatomical regions is scored with the highest AIS. The AIS values of the three most severely injured areas are squared and then summed.

Downloaded from http://injuryprevention.bmj.com/ on May 8, 2016 - Published by group.bmj.com

To reflect the physical reaction of the patient, the Glasgow Coma Scale, systolic blood pressure and respiratory rate are recorded at the moment the patient enters the ED. In addition, type (blunt or penetrating) and mechanism (eg, traffic, fall) of trauma will be collected from the trauma registry.

Comorbidity

To measure comorbidities, we will use a modified version of the Cumulative Illness Rating Scale (CIRS).[47] The CIRS is a valid instrument to use in hospitalised patients. In addition, the measure was found to be an indicator of health status and demonstrated its ability to predict 18-month mortality and rehospitalisation in hospitalised elderly patients.[48]

Data collection: follow-up questionnaires

Within the first week of hospital stay, patients will receive an information letter, informed consent form and the first questionnaire for the study either at the hospital or sent by post to patients home address.

Patients will be asked if they prefer to fill in the questionnaires online or by paper and pencil in the future. Returned questionnaires do not contain names or other overt identifiers, but are coded by number to link with the collected study data. Data will be collected by self-reported questionnaires at 1 week, and 1, 3, 6, 12 and 24 months after injury. See table 1 for an overview of the measures and measure moments.

Proxy informants have to sign an informed consent form for proxies before participating in the study. Proxies will enrol in the study for the 1-month (2nd) questionnaire. They will receive a shorter and customised questionnaire since not all instruments can be filled out by proxy informants (see table 1).

Severely injured patients (ISS>15) and patients with moderate-to-severe brain injury (AIS≥3) will receive a structured interview with the Glasgow Outcome Scale Extended (GOS-E) besides the standard set of questionnaires. In patients with brain injury (AIS≥3), the Quality of Life after Brain Injury Overall Scale (QOLIBRI-OS) will be administered as well. The structured interview will be performed during regular visits to the outpatient clinic or during consultation by telephone.

We will administer the following questionnaires:

- *EuroQol-5D (EQ-5D)*[49] to measure generic HRQoL. In the EQ-5D, health is defined along five dimensions; mobility, self-care, usual activities, pain or discomfort, and anxiety or depression. Each dimension has three levels: no problems, moderate problems or severe problems. A scoring algorithm is available by which each health status description can be expressed into a summary score. This summary score ranges from 0 for death and 1 for full health, and can be interpreted as a judgement on the relative desirability of a health status compared with perfect health. The standard EQ-5D classification does not include cognitive disability. Therefore, one item was added on cognition ("I have no/some/extreme problems with cognitive function, eg, memory, concentration, coherence, IQ").[49] According to the review of Polinder *et al*,[16] the EQ-5D has been used in various studies measuring HRQoL in trauma patients.
- *Health Utilities Index (HUI)*[50] to measure generic HRQoL. The HUI is a self-administered health status questionnaire that consists of 15 questions, which classifies respondents into either the HUI Mark 2 (HUI2) or the HUI Mark 3 (HUI3) health states. It covers the main health domains that are affected by injury, with particular focus on functional capacities. Results of the questionnaires are converted by an algorithm into the levels of the complementary HUI2 and HUI3 classification system to form seven-element and eight-element health state vectors. From these vectors, single-attribute and overall health state utility scores are calculated.[50] The HUI2 and the HUI3 have been used in a large variety of clinical studies[51] and have been used in two recent studies[52 53] in which trauma patients were involved. Furthermore, Polinder *et al*[53] and Van Beeck *et al*[54] stated that a combination of the EQ-5D and the HUI should be used in trauma patients since the combination of both measures covers all relevant dimensions of health.
- *Hospital Anxiety and Depression Scale (HADS)*,[55] a self-reported 14-item questionnaire to screen for anxiety and depressive disorders. Both types of disorders are assessed with seven questions. The HADS has a four-point response scale (0–3) and subscale scores range from 0 to 21. Subscale values ≥11 for one of the subscales were regarded as a psychological complaint as this cut-off score provides the lowest proportion of false positives (1% for depression and 5% for anxiety).[55] In 2009, the HADS has been validated as a screening tool for depression and anxiety in patients with TBI.[56] The HADS has been used in various studies including trauma patients.[57–59]
- *Impact Event Scale (IES)*[60] to measure symptoms of PTSD. The IES is a 15-item self-report questionnaire that measures intrusive re-experiences of the trauma and avoidance of trauma-related stimuli.[61] The respondent states whether the content of each statement was present using a four-point scale—0 (not at all), 1 (rarely), 3 (sometimes) or 5 (often)—during the past seven days. The total score of the IES ranges from 0 to 75, a score of at least 35 represents the best cut-off for a probable diagnosis of PTSD.[60] The IES has been widely used as a measure of stress reaction after a traumatic event, and this questionnaire is able to discriminate between stress reactions at different times after the event. Furthermore, the IES has good convergent validity with observer-diagnosed PTSD.[62] The IES has been used in earlier studies that included a population of trauma patients.[58 63]
- iMTA Medical Consumption Questionnaire (*i*MCQ)[64] is a recently developed non-disease-specific instrument for measuring (direct) medical costs. The *i*MCQ includes questions related to frequently occurring contacts with healthcare providers. The instrument is a standardised self-reported questionnaire and consists of 31 questions. The questions are based on the Dutch healthcare system. The *i*MCQ can be adapted for specific study populations and can be complemented with extra questions that are relevant for specific study populations.[64–66]
- iMTA Productivity Cost Questionnaire (*i*PCQ)[67] is a recently developed non-disease-specific self-report questionnaire and is applicable to national and international studies. Currently, a Dutch and an English version of the *i*PCQ are available. The *i*PCQ includes 18 questions. As in the case of the *i*MCQ, the *i*PCQ can be adapted for specific study populations and can be complemented with extra questions that are relevant for specific study populations. Both indirect costs due to absenteeism as the productivity losses (ie, presenteeism: sick, but working) are taken into account.[67] The questions of the *i*PCQ are based on the Short–Form Health and Labour Questionnaire (SF-HLQ),[68] the PROductivity and DISease Questionnaire[69] and the QQ method.[70] One question of the SF-HLQ will be added, a question about the cause(s) of reduced work capacity (eg, concentration problems). Furthermore, two questions about preinjury working status will be added. The *i*MCQ and the *i*PCQ have a similar

Table 1 Overview of the measures of the Brabant Injury Outcome Surveillance study

			Measure points after injury							
			<1 week		1 month					
	Included patients		Pre injury	Post injury	Pre injury	Post injury	3 months	6 months	12 months	24 months
Socio-demographic	All	Patient		x						
		Proxy				x				
Mod. CIRS	All	Patient		x					x*	
		Proxy				x			x*	
EQ-5D	All	Patient	x	x		x	x	x	x	x
		Proxy			x	x	x	x	x	x
HUI2 Q3/HUI3 Q6 (emotional well-being)	All	Patient	x							
		Proxy			x					
GFI	≥65 years	Patient	x						x	
		Proxy			x				x	
HUI3 Q4 (use of walking aid)	≥65 years	Patient	x						x	
		Proxy			x				x	
HUI2/3	All	Patient		x		x	x	x	x	x
		Proxy				x	x	x	x	x
HADS	All	Patient		x		x	x	x	x	x
		Proxy								
IES	All	Patient		x		x	x	x	x	x
		Proxy								
*i*MCQ	All	Patient				x	x	x	x	x
		Proxy				x	x	x	x	x
*i*PCQ	All	Patient				x	x	x	x	x
		Proxy				x	x	x	x	x
GOS-E	Severely injured (ISS >15) and/or TBI (AIS ≥3)	Patient				x	x	x	x	x
		Proxy				x	x	x	x	x
QOLIBRI-OS	TBI (AIS ≥3)	Patient				x	x	x	x	x
		Proxy				x	x	x	x	x

*Only ≥65 years.
EQ-5D, EuroQol-5D; GFI, Groningen Frailty Index; GOS-E, Glasgow Outcome Scale Extended; HADS, Hospital and Anxiety Depression Scale; HUI, Health Utilities Index; IES, Impact Event Scale; *i*MCQ, iMTA Medical Consumption Questionnaire; *i*PCQ, iMTA Productivity Cost Questionnaire; Mod. CIRS, Modified Cumulative Illness Rating Scale; QOLIBRI-OS, Quality of Life after Brain Injury Overall Scale; TBI, traumatic brain injury.

Downloaded from http://injuryprevention.bmj.com/ on May 8, 2016 - Published by group.bmj.com

Downloaded from http://injuryprevention.bmj.com/ on May 8, 2016 - Published by group.bmj.com

structure and can be combined to measure productivity losses (direct and indirect costs) in detail.[65 67]

- *GOS-E*[71] to measure functional outcome in patients with moderate-to-severe TBI (AIS ≥3) and in severely injured patients (ISS score >15). The GOS-E consists of eight questions covering consciousness, independence at home, major social roles (work, social and leisure activities, family and friendships) and return to normal life.[33] It results in an eight-point scale classifying functional outcome from 1 (dead) to 8 (complete recovery). The GOS-E is a valid measure and is sensitive to change in patients with mild-to-moderate TBI.[72] The GOS-E is frequently used to measure functional outcome in patients with TBI.[73–77]

Originally, the GOS-E was developed for measuring head injury outcomes. However, as it includes most domains from the WHO's International Classification of Functioning, Disability and Health,[78] the GOS-E is recommended for use in a trauma population. The GOS-E is considered a responsive measure in non-head-injured patients.[79] To analyse the GOS-E outcomes of the severely injured patients, we will use the same dichotomised outcomes as stated in the study of Gabbe *et al;*[30] a score of ≥7 represents 'good recovery', whereas a score <7 represents 'poor recovery'. GOS-E scores will be determined using a standard structured interview.[71]

- *QOLIBRI-OS*[80] to measure HRQoL in patients with moderate-to-severe TBI (AIS ≥3). The QOLIBRI-OS is a recently developed measure and consists of six statements that cover areas including physical conditioning, cognition, emotions, function in daily life, personal and social life, and current situation and future prospects. Response to each item will be scored 1 ('not at all') to 5 ('very'). The sum score of the QOLIBRI-OS can be converted arithmetically to a percentage scale; 0 represents the lowest possible HRQoL, whereas 100 represents the best possible HRQoL.[80] The QOLIBRI-OS is a short version of the 37-item QOLIBRI scale and it assesses a similar construct to the QOLIBRI total score. The QOLIBRI-OS is considered a reliable and valid measure.[81 82]
- *Groningen Frailty Index (GFI)*[83] is a 15-item self-reported instrument to measure frailty. Frailty is defined as "a clinically recognizable state of increased vulnerability resulting from ageing-associated decline in reserve and function across multiple physiologic systems such that the ability to cope with every day or acute stressors is comprised".[84] Because we expect that frailty is a strong predictor in outcome after trauma, we will measure frailty in all patients aged 65 and older. The GFI screens for the loss of functions and resources in four domains of functioning: physical, cognitive, social and psychological.[83 85] The sum score of the GFI ranges from 0 to 15, with a score of ≥4 indicating frailty. The study of Peters *et al*[86] concluded that the GFI is a feasible, reliable and valid self-assessment in home-dwelling and institutionalised elderly people.

Preinjury and normative cohort data

Patients will be asked to fill in the EQ-5D and two questions about emotional well-being of the HUI (HUI2 question 3 and HUI3 question 6) for the preinjury status during the first one-week questionnaire, and proxy's during the second questionnaire (ie, 1 month after injury). Patients 65 years and older will be asked to fill out one question of the HUI (HUI3 question 4) to determine patients' level of ambulation and the need of a walking aid preinjury.

To examine differences in outcomes of the preinjury health status of our study population compared with a comparable Dutch population, a reference cohort of 1500 healthy individuals will be asked to fill out the same set of questionnaires as the included patients of our study measuring their preinjury health status. We will make use of the data of the Longitudinal Internet Studies for the Social sciences (LISS) panel administered by CentERdata (Tilburg University, The Netherlands). It is known that adult hospitalised trauma patients are not a representative sample of the general population since the trauma study population differs regarding age, gender and socioeconomic status.[87–89] By using the LISS panel, we will adjust for these variables. The normative cohort data will be a useful tool, in which results can be compared with the BIOS results.

Healthcare consumption and costs

Costs will include direct intramural and extramural medical costs, and indirect costs following absenteeism or presenteeism from work. The economic evaluation will be performed from a societal perspective in accordance with the Dutch guidelines.[90]

Direct intramural medical costs due to treatment, complications and events during follow-up (eg, ED visit, diagnostic work-up, therapy, surgery, admissions, follow-up visits) will be calculated. Real medical costs will be calculated by multiplying the volumes of healthcare consumption with the corresponding unit prices. All intramural activities registered after trauma will be obtained from the financial department of the hospital. We will use the unit prices determined by the financial department of the hospital, which are based on a detailed inventory and measurement of all resources used. For instance, the calculation of the costs of surgical procedures and hospital stay will consist of detailed measurement of investments in manpower, equipment, materials, housing and overhead.

Data on patients' extramural medical costs will be collected 1, 3, 6, 12 and 24 months post injury by using the *i*MCQ. Last, indirect costs due to productivity loss will be calculated based on information on work absence and return to work. Information will be collected 1, 3, 6, 12 and 24 months using the *i*PCQ. Different methods exist to value productivity. The well-known human-capital method takes the patient's perspective and counts any hour not worked as an hour lost.[91] By applying wage costs, the results of the *i*PCQ can be monetised and as such used in health economic evaluations.

Response rate

We will use some practical approaches to maximise the response rate. First of all, we will use prepaid reply envelopes. Second, all patients will be contacted by telephone by the research employees within 1 week after trauma on behalf of the participating hospitals. Third, patients can choose to fill in the questionnaires electronically or by paper and pencil. If necessary, we will send reminders with second copies of the questionnaires. Fourth, patients can still flow in into the study at 1 or 3 months after trauma.

In the BIOS study, we will investigate the injuries of a representative part of the Netherlands. Of all patients included in the Dutch Trauma Registry, 16% is admitted to 1 of the 10 hospitals of the region Noord-Brabant.[34]

About 12 000 trauma patients are admitted in the Brabant region annually. Assuming 2000 patients do not meet the inclusion criteria (deceased in hospital or age <18), 10 000 patients can be recruited for the study.

 de Jongh MAC, *et al*. *Inj Prev* 2016;**0**:1–9. doi:10.1136/injuryprev-2016-042032

Downloaded from http://injuryprevention.bmj.com/ on May 8, 2016 - Published by group.bmj.com

Data analysis

All analyses will be conducted using SPSS V.19.0 (Statistical Package for Social Sciences, Chicago, Illinois, USA).

Frequencies and descriptive statistics will be calculated to provide an overview of the characteristics of the study population. Statistical test results will be considered significant at a level of $p<0.05$. Student's t test and one-way analysis of variance will be used to compare continuous variables. χ^2 tests will be performed for nominal variables. Mixed models will be used to examine the course of HRQoL, functional, psychological and societal outcome over time and between different groups. Missing values will be imputed according to the guidelines of the questionnaires. Socio-demographic, psychological and injury-related characteristics will be tested as risk factors of decreased HRQoL, functional, psychological and societal outcome and increased costs measured 1, 3, 6, 12 and 24 months after injury in simple and multiple regression analysis. Regarding the work ability after trauma, we will conduct survival analyses. The results of the proxy informants will be analysed separately.

Prediction model

For the prediction model of non-fatal outcome, we will use the data collected in the prospective study. Correlation between the different non-fatal outcome measures will be calculated with Spearman's rho test. Predictors for non-fatal outcome are assessed using stepwise multiple regression models. The performance of the models will be assessed in terms of calibration and discrimination. The validity of the final model will be tested. The role and effect of MI will be investigated.

FUTURE PERSPECTIVE

The BIOS study with a relatively large sample size, measurement of preinjury and short-term and long-term functional outcomes and a wide range of outcome measures should constitute a detailed and comprehensive study of non-fatal injuries of varying severity. The focus on non-fatal outcomes and morbidity is critical as the burden of disability on society substantially outweighs the burden of mortality. The methodological developments and data from this study should also make a substantial contribution to the international collaborative effort to assess the societal impact and burden of injuries more accurately.

In traditional evaluation studies, observed and expected mortality are compared to assess quality of care. Regarding the increased survival rates, other outcome models are required to assess and improve the quality of trauma care. In our opinion, these models have to include fatal outcome, non-fatal outcome measures and costs. Little is known about the interaction between the different outcome aspects. Furthermore, it is plausible that predictors and scores in non-fatal outcome models are different from the classical fatal outcome models. As far as we know, models for non-fatal outcome on different aspects for a complete clinical trauma population have never been developed. The BIOS study results will be used to build a new model including fatal and non-fatal outcome.

The BIOS study will be a building block model with a base data set and opportunities to enlarge with specific data or questionnaires for specific injuries. For example, patients with a hip fracture and aged >65 are receiving extra questionnaires specific for quality of life within the elderly and functioning and pain after a hip fracture. Another example, patients with an acetabular fracture will be asked to complete the modified Merle d'Aubigne hip score[92] together with a medical expert during a standard visit to the outpatient clinic, next to the BIOS questionnaire. Furthermore, patients with a pelvic fracture will also be asked to complete the Majeed pelvic score.[93]

One of the aims of this project is to investigate whether an enlargement of the trauma registry with patient-reported outcome measurement does add value. A part of this aim will be to define which questionnaires and data should be collected structurally.

The findings of the proposed BIOS study will have significant benefits for understanding the impact of non-fatal injury on personal and population health. This consistently collected empirical data will support the production of more valid burden-of-injury calculations, differences in outcomes and burden experienced by injury subgroups, cost-effectiveness analyses of injury prevention programmes and trauma care, and support continuous quality improvement of care.

Collaborators *Members of the BIOS group*: P V van Eerten, F C van Eijck, H J A A van Geffen, W A J J M Haagh, L M S J Poelhekke, J B Sintenie, C T Stevens, A H van der Veen, C H van der Vlies and D I Vos.

Contributors All authors contributed to the paper.

Funding ZonMw (80-84200-98-14255).

Competing interests None declared.

Ethics approval Medical Ethics Committee Brabant (project number NL50258.028.14).

Provenance and peer review Not commissioned; internally peer reviewed.

REFERENCES

1 Polinder S, Meerding WJ, Mulder S, *et al*. Assessing the burden of injury in six European countries. *Bull World Health Organ* 2007;85:27–34.
2 Polinder S, Meerding WJ, van Baar ME, *et al*. Cost estimation of injury-related hospital admissions in 10 European countries. *J Trauma* 2005;59:1283–91.
3 MacKenzie EJ, Rivara FP, Jurkovich GJ, *et al*. A national evaluation of the effect of trauma-center care on mortality. *N Engl J Med* 2006;354:366–78.
4 Vles WJ, Steyerberg EW, Essink-Bot ML, *et al*. Prevalence and determinants of disabilities and return to work after major trauma. *J Trauma* 2005;58:126–35.
5 Holtslag HR, van Beeck EF, Lichtveld RA, *et al*. Individual and population burdens of major trauma in the Netherlands. *Bull World Health Organ* 2008;86:111–17.
6 Polinder S, Haagsma JA, Lyons RA, *et al*. Measuring the population burden of fatal and nonfatal injury. *Epidemiol Rev* 2012;34:17–31.
7 Bhalla K, Harrison J, Abraham J, *et al*. Data sources for improving estimates of the global burden of injuries: call for contributors. *PLoS Med* 2009;6:e1.
8 Polinder S, van Beeck EF, Essink-Bot ML, *et al*. Functional outcome at 2.5, 5, 9, and 24 months after injury in the Netherlands. *J Trauma* 2007;62:133–41.
9 Kendrick D, Vinogradova Y, Coupland C, *et al*. Recovery from injury: the UK burden of injury multicentre longitudinal study. *Inj Prev* 2013;19:370–81.
10 Langley J, Derrett S, Davie G, *et al*. A cohort study of short-term functional outcomes following injury: the role of pre-injury socio-demographic and health characteristics, injury and injury-related healthcare. *Health Qual Life Outcomes* 2011;9:68.
11 Derrett S, Langley J, Hokowhitu B, *et al*. Prospective outcomes of injury study. *Inj Prev* 2009;15:e3.
12 Mackenzie EJ, Rivara FP, Jurkovich GJ, *et al*. The national study on costs and outcomes of trauma. *J Trauma* 2007;63(6 Suppl):S54–67; discussion S81-6.
13 Edwards ER, Graves SE, McNeil JJ, *et al*. Orthopaedic trauma: establishment of an outcomes registry to evaluate and monitor treatment effectiveness. *Injury* 2006;37:95–6.
14 Lyons RA, Towner EE, Kendrick D, *et al*. The UK burden of injury study—a protocol. [National Research Register number: M0044160889. *BMC Public Health* 2007;7:317.
15 Gabbe BJ, Braaf S, Fitzgerald M, *et al*. RESTORE: REcovery after Serious Trauma-Outcomes, Resource use and patient Experiences study protocol. *Inj Prev* 2015;21:348–54.
16 Polinder S, Haagsma JA, Belt E, *et al*. A systematic review of studies measuring health-related quality of life of general injury populations. *BMC Public Health* 2010;10:783.
17 van Delft-Schreurs CC, van Bergen JJ, van de Sande P, *et al*. A cross-sectional study of psychological complaints and quality of life in severely injured patients. *Qual Life Res* 2014;23:1353–62.
18 Nichol AD, Higgins AM, Gabbe BJ, *et al*. Measuring functional and quality of life outcomes following major head injury: common scales and checklists. *Injury* 2011;42:281–7.

Downloaded from http://injuryprevention.bmj.com/ on May 8, 2016 - Published by group.bmj.com

19 Shi Q, Sinden K, MacDermid JC, *et al*. A systematic review of prognostic factors for return to work following work-related traumatic hand injury. *J Hand Ther* 2014;27:55–62.
20 Utomo WK, Gabbe BJ, Simpson PM, *et al*. Predictors of in-hospital mortality and 6-month functional outcomes in older adults after moderate to severe traumatic brain injury. *Injury* 2009;40:973–7.
21 van Erp S, Holtslag HR, van Beeck EF. Determinants of limitations in unpaid work after major trauma: aprospective cohort study with 15 months follow-up. *Injury* 2014;45:629–34.
22 Gabbe BJ, Simpson PM, Sutherland AM, *et al*. Evaluating time points for measuring recovery after major trauma in adults. *Ann Surg* 2013;257:166–72.
23 Holtslag HR, Post MW, Lindeman E, *et al*. Long-term functional health status of severely injured patients. *Injury* 2007;38:280–9.
24 Holtslag HR, Post MW, van der Werken C, *et al*. Return to work after major trauma. *Clin Rehabil* 2007;21:373–83.
25 Haagsma JA, Polinder S, Olff M, *et al*. Posttraumatic stress symptoms and health-related quality of life: a two year follow up study of injury treated at the emergency department. *BMC Psychiatry* 2012;12:1.
26 van Delft-Schreurs CC, van Bergen JJ, de Jongh MA, *et al*. Quality of life in severely injured patients depends on psychosocial factors rather than on severity or type of injury. *Injury* 2014;45:320–6.
27 Baranyi A, Leithgöb O, Kreiner B, *et al*. Relationship between posttraumatic stress disorder, quality of life, social support, and affective and dissociative status in severely injured accident victims 12 months after trauma. *Psychosomatics* 2010;51:237–47.
28 Sutherland AG, Alexander DA, Hutchison JD. The mind does matter: psychological and physical recovery after musculoskeletal trauma. *J Trauma* 2006;61:1408–14.
29 Kennedy RL, Grant PT, Blackwell D. Low-impact falls: demands on a system of trauma management, prediction of outcome, and influence of comorbidities. *J Trauma* 2001;51:717–24.
30 Gabbe BJ, Simpson PM, Lyons RA, *et al*. Association between the number of injuries sustained and 12-month disability outcomes: evidence from the Injury-VIBES Study. *PLoS ONE* 2014;9:e113467.
31 Makary MA, Segev DL, Pronovost PJ, *et al*. Frailty as a predictor of surgical outcomes in older patients. *J Am Coll Surg* 2010;210:901–8.
32 Bakker FC, Olde Rikkert MG. Hospital Care for Frail Elderly Adults: From Specialized Geriatric Units to Hospital-Wide Interventions. *Interdiscip Top Gerontol Geriatr* 2015;41:95–106.
33 Kim SW, Han HS, Jung HW, *et al*. Multidimensional frailty score for the prediction of postoperative mortality risk. *JAMA Surg* 2014;149:633–40.
34 Traumazorg in beeld: Landelijke Traumaregistratie 2010-2014, rapportage Nederland: Landelijk Netwerk Acute Zorg, 2015.
35 Bergeron E, Rossignol M, Osler T, *et al*. Improving the TRISS methodology by restructuring age categories and adding comorbidities. *J Trauma* 2004;56:760–7.
36 Determination of national normative outcomes for trauma. Journals of trauma-injury infection and critical care: Williams & Wilkins 351 West Camden St, Baltimore, MD, 21201-2436; 1984.
37 Lefering R, Huber-Wagner S, Nienaber U, *et al*. Update of the trauma risk adjustment model of the TraumaRegister DGU™: the Revised Injury Severity Classification, version II. *Crit Care* 2014;5:476.
38 Jones JM, Skaga NO, Søvik S, *et al*. Norwegian survival prediction model in trauma: modelling effects of anatomic injury, acute physiology, age, and co-morbidity. *Acta Anaesthesiol Scand* 2014;58:303–15.
39 Boyd CR, Tolson MA, Copes WS. Evaluating trauma care: the TRISS method. Trauma Score and the Injury Severity Score. *J Trauma* 1987;27:370–8.
40 Champion HR, Sacco WJ, Carnazzo AJ, *et al*. Trauma score. *Crit Care Med* 1981;9:672–6.
41 Baker SP, O'Neill B, Haddon W Jr, *et al*. The injury severity score: a method for describing patients with multiple injuries and evaluating emergency care. *J Trauma* 1974;14:187–96.
42 Baker SP, O'neill B. The injury severity score: an update. *J Trauma* 1976;16:882–5.
43 Hukkelhoven CW, Steyerberg EW, Habbema JDF, *et al*. Predicting outcome after traumatic brain injury: development and validation of a prognostic score based on admission characteristics. *J Neurotrauma* 2005;22:1025–39.
44 Steyerberg EW, Mushkudiani N, Perel P, *et al*. Predicting outcome after traumatic brain injury: development and international validation of prognostic scores based on admission characteristics. *PLoS Med* 2008;5:e165.
45 Gennarelli TA, Wodzin E. AIS 2005: a contemporary injury scale. *Injury* 2006;37:1083–91.
46 Committee on Injury Scaling, Association for the Advancement of Automotive Medicine (AAAM). The Abbreviated Injury Scale, 1990 Revision. 2008.
47 Linn BS, LINN MW, Gurel L. Cumulative illness rating scale. *J Am Geriatr Soc* 1968;16:622–6.
48 Salvi F, Miller MD, Grilli A, *et al*. A manual of guidelines to score the modified cumulative illness rating scale and its validation in acute hospitalized elderly patients. *J Am Geriatr Soc* 2008;56:1926–31.
49 The EuroQol Group. EuroQol-a new facility for the measurement of health-related quality of life. *Health Policy* 1990;16:199–208.
50 Furlong W, Feeny D, Torrance G. *Health Utilities Index (HUI) procedures manual: algorithm for determining HUI Mark 2 (HUI2)/Mark 3 (HUI3) health status classification levels, health states single-attribute level utility scores and overall health-related quality of life utility scores from 15-item self-complete health status questionnaires*. Hamilton, ON: Health Utilities Inc, 2000.
51 Furlong WJ, Feeny DH, Torrance GW, *et al*. The Health Utilities Index (HUI®) system for assessing health-related quality of life in clinical studies. *Ann Med* 2001;33:375–84.
52 Ringburg AN, Polinder S, van Ierland MC, *et al*. Prevalence and prognostic factors of disability after major trauma. *J Trauma* 2011;70:916–22.
53 Polinder S, Haagsma JA, Bonsel G, *et al*. The measurement of long-term health-related quality of life after injury: comparison of EQ-5D and the health utilities index. *Inj Prev* 2010;16:147–53.
54 Van Beeck EF, Larsen CF, Lyons RA, *et al*. Guidelines for the conduction of follow-up studies measuring injury-related disability. *J Trauma* 2007;62:534–50.
55 Zigmond AS, Snaith RP. The hospital anxiety and depression scale. *Acta Psychiatr Scand* 1983;67:361–70.
56 Whelan-Goodinson R, Ponsford J, Schönberger M. Validity of the Hospital Anxiety and Depression Scale to assess depression and anxiety following traumatic brain injury as compared with the Structured Clinical Interview for DSM-IV. *J Affect Disord* 2009;114:94–102.
57 Bocci MG, Grieco DL, Lochi S, *et al*. Defining needs and goals of post-ICU care for trauma patients: preliminary study. *Minerva Anestesiol* 2016;82:22–9.
58 Whittaker R, Kemp S, House A. Illness perceptions and outcome in mild head injury: a longitudinal study. *J Neurol Neurosurg Psychiatr* 2007;78:644–6.
59 O'Donnell ML, Varker T, Holmes AC, *et al*. Original research disability after injury: the cumulative burden of physical and mental health. *J Clin Psychiatry* 2013;74:e137–43.
60 Wohlfarth TD, van den Brink W, Winkel FW, *et al*. Screening for Posttraumatic Stress Disorder: an evaluation of two self-report scales among crime victims. *Psychol Assess* 2003;15:101.
61 van der Ploeg E, Mooren TT, Kleber RJ, *et al*. Construct validation of the Dutch version of the impact of event scale. *Psychol Assess* 2004;16:16.
62 Sundin EC, Horowitz MJ. Impact of Event Scale: psychometric properties. *Br J Psychiatry* 2002;180:205–9.
63 Perry S, Difede J, Musngi G, *et al*. Predictors of posttraumatic stress disorder after burn injury. *Am J Psychiatry* 1992;149:931–5.
64 Bouwmans C, Hakkaart-van Roijen L, Koopmanschap M, *et al*. *Handleiding iMTA Medical Costs Questionnaire*. Rotterdam: Institute for Medical Technology Assessment—Erasmus Universiteit Rotterdam, 2013.
65 http://www.bmg.eur.nl/english/imta/publications/questionnaires_manuals/ (accessed 21 Nov 2015).
66 Bouwmans CAM. Medical Consumption Questionnaire, Producitivy and Health Research Group, Handleiding iMTA Medical Consumption Questionnaire, 2013.
67 Bouwmans C, Hakkaart-van Roijen L, Koopmanschap M, *et al*. *Handleiding iMTA Productivity Costs Questionnaire (iPCQ)*. Rotterdam: Institute for Medical Technology Assessment—Erasmus Universiteit Rotterdam, 2013.
68 Van Roijen L, Essink-Bot ML, Koopmanschap MA, *et al*. Labor and health status in economic evaluation of health care: The Health and Labor Questionnaire. *Int J Technol Assess Health Care* 1996;12:405–15.
69 Koopmanschap MA. PRODISQ: a modular questionnaire on productivity and disease for economic evaluation studies. *Expert Rev Pharmacoecon Outcomes Res* 2005;5:23–8.
70 Brouwer WB, Koopmanschap M, Rutten F. Productivity losses without absence: measurement validation and empirical evidence. *Health Policy* 1999;48:13–27.
71 Wilson JL, Pettigrew LE, Teasdale GM. Structured interviews for the Glasgow Outcome Scale and the extended Glasgow Outcome Scale: guidelines for their use. *J Neurotrauma* 1998;15:573–85.
72 Levin HS, Boake C, Song J, *et al*. Validity and sensitivity to change of the extended Glasgow Outcome Scale in mild to moderate traumatic brain injury. *J Neurotrauma* 2001;18:575–84.
73 Sandhaug M, Andelic N, Langhammer B, *et al*. Functional level during the first 2 years after moderate and severe traumatic brain injury. *Brain Inj* 2015;29:1431–8.
74 Scheenen ME, de Koning ME, van der Horn, *et al*. Acute alcohol intoxication in patients with mild traumatic brain injury: characteristics, recovery, and outcome. *J Neurotrauma* 2015;33:339–45.
75 Alway Y, McKay A, Gould KR, *et al*. Factors associated with posttraumatic stress disorder following moderate to severe traumatic brain injury: a prospective study. *Depress Anxiety* 2016;33:19–26.
76 Drake AI, McDonald EC, Magnus NE, *et al*. Utility of Glasgow Coma Scale-Extended in symptom prediction following mild traumatic brain injury. *Brain Inj* 2006;20:469–75.

Downloaded from http://injuryprevention.bmj.com/ on May 8, 2016 - Published by group.bmj.com

77 Anke A, Andelic N, Skandsen T, *et al*. Functional recovery and life satisfaction in the first year after severe traumatic brain injury: a prospective multicenter study of a Norwegian national cohort. *J Head Trauma Rehabil* 2015;30:E38–49.
78 Ardolino A, Sleat G, Willett K. Outcome measurements in major trauma—results of a consensus meeting. *Injury* 2012;43:1662–6.
79 Williamson OD, Gabbe BJ, Sutherland AM, *et al*. Comparing the responsiveness of functional outcome assessment measures for trauma registries. *J Trauma* 2011;71:63–8.
80 von Steinbuechel N, Wilson L, Gibbons H, *et al*. QOLIBRI overall scale: a brief index of health-related quality of life after traumatic brain injury. *J Neurol Neurosurg Psychiatry* 2012;83:1041–7.
81 von Steinbuechel N. Lessons From the Qolibri Overall Scale (QOLIBRI-OS) for its use Worldwide. *Eur Health Psychol* 2014;16(Suppl):642.
82 von Steinbüchel N, Wilson L, Gibbons H, *et al*. Quality of Life after Brain Injury (QOLIBRI): scale validity and correlates of quality of life. *J Neurotrauma* 2010;27:1157–65.
83 Schuurmans H, Steverink N, Lindenberg S, *et al*. Old or frail: what tells us more? *J Gerontol A Biol Sci Med Sci* 2004;59:M962–5.
84 Xue QL. The frailty syndrome: definition and natural history. *Clin Geriatr Med* 2011;27:1–15.
85 Steverink N, Slaets J, Schuurmans H, *et al*. Measuring frailty: developing and testing the GFI (Groningen frailty indicator). *Gerontologist* 2001;41:236–7.
86 Peters LL, Boter H, Buskens E, *et al*. Measurement properties of the Groningen Frailty Indicator in home-dwelling and institutionalized elderly people. *J Am Med Dir Assoc* 2012;13:546–51.
87 Moore L, Turgeon AF, Sirois MJ, *et al*. Influence of socioeconomic status on trauma center performance evaluations in a Canadian trauma system. *J Am Coll Surg* 2011;213:402–9.
88 Hanna CL, Hasselberg M, Laflamme L, *et al*. Road traffic crash circumstances and consequences among young unlicensed drivers: a Swedish cohort study on socioeconomic disparities. *BMC Public Health* 2010;10:14.
89 Hasselberg M, Laflamme L. Socioeconomic background and road traffic injuries: a study of young car drivers in Sweden. *Traffic Inj Prev* 2003;4:249–54.
90 Tan SS, Bouwmans-Frijters CA, Hakkaart-van Roijen L. Handleiding voor kostenonderzoek: methoden en referentieprijzen voor economische evaluaties in de gezondheidszorg. *Tijdschrift voor gezondheidswetenschappen* 2012;90:367–72.
91 Dagum C. *Human capital*. Encyclopedia of Statistical Sciences, 2004.
92 D'Aubigne RM, Postel M. Functional results of hip arthroplasty with acrylic prosthesis. *J Bone Joint Surg Am* 1954;36-A:451–475.
93 Majeed SA. Grading the outcome of pelvic fractures. *J Bone Joint Surg Br* 1989;71:304–6.

Downloaded from http://injuryprevention.bmj.com/ on May 8, 2016 - Published by group.bmj.com

IP

Prevalence, recovery patterns and predictors of quality of life and costs after non-fatal injury: the Brabant Injury Outcome Surveillance (BIOS) study

M A C de Jongh, N Kruithof, T Gosens, C L P van de Ree, L de Munter, L Brouwers, S Polinder and K W W Lansink

Inj Prev published online May 6, 2016

Updated information and services can be found at:
http://injuryprevention.bmj.com/content/early/2016/05/06/injuryprev-2016-042032

These include:

References

This article cites 84 articles, 11 of which you can access for free at:
http://injuryprevention.bmj.com/content/early/2016/05/06/injuryprev-2016-042032#BIBL

Email alerting service

Receive free email alerts when new articles cite this article. Sign up in the box at the top right corner of the online article.

Topic Collections

Articles on similar topics can be found in the following collections

Epidemiologic studies (837)

Notes

To request permissions go to:
http://group.bmj.com/group/rights-licensing/permissions

To order reprints go to:
http://journals.bmj.com/cgi/reprintform

To subscribe to BMJ go to:
http://group.bmj.com/subscribe/
