# Supplementary material for: Prognostic factors and quality of life after pelvic fractures. The Brabant Injury Outcome Surveillance (BIOS) study
Source: PLoS One. 2020 Jun 11;15(6):e0233690. doi: 10.1371/journal.pone.0233690 (PMC7289384; doi:10.1371/journal.pone.0233690)
Supplement: Attachment — Submitted filename: PLOS - review BIOS study 2020.docx [file pone.0233690.s002.docx]

Thank you for the opportunity to review this paper.

This is a prospective multicentre cohort observational study examining pelvic fracture impact on HRQoL for 12 months post injury utilising a series of scoring systems. The prospectively maintained database followed post-injury trauma patients with generic and injury specific QOLs. Th authors conclude that low pre-injury scores, female gender and higher ISS are prognostic for lower post-injury scores.

The authors should be commended on their work. There are a few areas that would need to be addressed before publication.

The exclusion criteria includes patients over 80. This is a very common age group to sustain low energy pelvic/acetabular injuries. What were the reasons for exclusion of this particular group?

The impact of concomitant injuries and their impact upon their outcome scores could use more exploration. Whilst stratified for ISS there seems little discussion on the obvious effect that other injuries would have upon patient outcomes.

The use of the retrospective pre-injury score is fraught with some difficulties as noted by the authors. It is noted to be a prognostic factor for low post-injury scores. It is however unclear if this is a globally reduced score, or whether it is a reduced score relative to their baseline. It is unclear whether this is calculated relative to their pre-injury scores or whether this is calculated on the mean (we would not expect their pelvic injury to increase their QoL scores!)

Is there any 24 month data? Its noted in the other BIOS article referenced ( Prevalence, recovery patterns and predictors of quality of life and costs after non-fatal injury: the Brabant Injury Outcome Surveillance (BIOS) study) That 24 month data points were collected. The authors have referenced 24 and 36 month outcomes from other papers, it would useful to include this data for comparison with the other studies referenced in the introduction.

Figure 3, only demonstrates a single data point? Should there be separate points for both <65 and >65? Visually a line graph connecting the data points would be easier to follow than dots.

As noted by the authors, pelvic fractures have a bimodality of injury presentation. Breakdown by high energy and low energy would be useful rather than purely fracture pattern type to highlight the two different disease patterns,

Similarly this would give more meaning to the presence/absence of concomitant injuries and the types of shock on presentation, absence and presence of surgery. Furthermore, indications of requirements for other non-pelvic surgery related to their trauma would be useful, especially in the conservatively managed multi-trauma pelvic fracture patient.
