# Supplementary material for: Prognostic factors and quality of life after pelvic fractures. The Brabant Injury Outcome Surveillance (BIOS) study
Source: PLoS One. 2020 Jun 11;15(6):e0233690. doi: 10.1371/journal.pone.0233690 (PMC7289384; doi:10.1371/journal.pone.0233690)
Supplement: Attachment — Submitted filename: Response_to_reviewers_Brouwers.PLOSONE.docx [file pone.0233690.s003.docx]

Zsolt J. Balogh, MD, PhD, FRACS

Academic Editor

PLOS ONE

May 5^th^ 2020

Dear dr. Balogh,

Subject: PONE-D-20-05544

We were very grateful for reviewing our manuscript. We think the comments of the reviewers have taken the manuscript to a next level. In the following pages we outlined our point-by-point responses to each of the comments of the reviewers.

We hope that the revisions in the manuscript will be sufficient to make our manuscript suitable for publication in PLOS ONE.

ZonMw stands for ZorgOnderzoek Nederland (Zon) en het gebied Medische wetenschappen (Mw). However, the organization use ZonMw to communicate: https://www.zonmw.nl/en/about-zonmw/organisation/.

Kind regards,

Lars Brouwers

**Comments reviewer 1**

Why did you include patients with acetabular fractures? As you already pointed out in the discussion patients with acetabular fractures have different issues that can influence HRQoL like osteoarthritis, heterotopic ossification and avascular necrosis of the femoral head. Would you consider a sub analysis of patients with acetabular fractures? If not, why not?

- Pelvic fractures represent a broad injury spectrum. Minor pelvic ring (and isolated acetabular) fracture are typically sustained by elderly patients following low-impact falls. Furthermore some patients have both types of fractures: for example Tile C and an acetabular fracture. By contrast, major high-energy pelvic ring disruptions may be life threatening and are largely found in young multiple-injured patients. We included both acetabular fractures and pelvic ring fractures, because we think pelvic fractures have a binary presentation: low- and high energy trauma. We felt these outcomes were more important for daily practice when compared with fractures types.

Our hypothesis was that the type of fracture is just as important as other prognostic factors. Therefore HRQoL outcome over time and prognostic factors in the first year were measured for both pelvic ring fractures and acetabular fractures (Table 4). We did perform a sub analysis for both types of pelvic fractures. No significant differences when comparing different subtypes of pelvic ring fractures of acetabular fractures were found (Table 5 and 6). Also, no significant difference was found between low- and high energy trauma for both acetabular- and pelvic fractures.

We agree with the reviewer, that the sub analysis could be better underlined in the discussion section. We included this section in the “prognostic factor” paragraph.

How do you explain that type pelvic type, acetabulum type, pelvic operation, high- or low-energy trauma were not significantly associated with lower or higher HRQoL after pelvic injury. It is difficult to understand that a patient with for example a type C pelvic fracture who underwent extensive surgery would have a similar HRQoL compared with a patient with conservatively treated type A pelvic fracture. In a paper published in Injury 2018 (ref 28) you conclude that Tile C patients <65 years had significantly lower EQ-5D index and total MPS scores. Please explain this inconsistency.

- Although injury type was not significantly associated with higher or lower HRQoL after pelvic injury, it could be possible that the sample size of patients was insufficient to draw conclusions about these prognostic factors. It could also be possible that prognostic factors such as pelvic or acetabular type and operation are of importance to the follow-up during the first months after trauma. Prognostic factors were assessed over the first year after injury; prognostic factors for short-term recovery could be leveled out if they were not also prognostic factors for long-term recovery. Therefore, a new study is needed with larger sample sizes to draw conclusions using these prognostic factors.
- We agree with the reviewer that this conclusion is inconsequent with our study published in 2018. However, in 2018 we performed a cross sectional analysis while in this paper a longitudinal analysis was used. We now feel that a longitudinal analysis is much better in investigating HRQoL patterns when compared with just one follow-up moment without adjustment for the pre-injury score. We underlined this in the discussion section.

You demonstrated that female gender was associated with a lower HRQoL, but age was not. In the discussion you state “we did not find a relationship between aging and a decreased HRQoL. An important reason for this lack of association could be the inclusion of a preinjury HRQoL questionnaire in our longitudinal analysis, which was not included in the questionnaires of other studies”. Since elderly patients were more likely female, how do you explain that age was not a prognostic factor. Is it possible that females had lower preinjury HRQoL? Please explain

- As stated in our study; Polinder^1^ and Holbrook^2^ concluded a better understanding in the difference between the impact in men and women is needed to improve trauma care.

Mean age of females in our study was 62 (SD 16) years and mean age for male patients was 56 (SD 17) years (P = 0.03). Mean pre-injury EQ5D index score of female patients was 0.85 (SD 0.20) while the score of male patients was 0.93 (SD 0.16), a significant difference (P = 0.01), indicating lower pre-injury health status for females compared to males.

We included the univariable mixed models analyses in supplemental tables to this point-by-point letter. These analyses showed that age is not a prognostic factor in univariable analyses, which is in accordance with the multivariable analyses. This explains that baseline condition of the patient is of more importance for predicting outcome, compared to the age of the patient. Females showed to have lower pre-injury health status. Univariable, both factors, gender and pre-injury health status, are prognostic factors and also in some of the multivariable analyses both factors showed to be significantly associated with outcome after pelvic injury. We included this in our manuscript in the results and discussion section.

1. Polinder S, van Beeck EF, Essink-Bot ML, Toet H, Looman CW, Mulder S, et al. Functional outcome at 2.5, 5, 9, and 24 months after injury in the Netherlands. Journal of Trauma and Acute Care Surgery 2007;62(1):133-141
2. Holbrook TL, Anderson JP, Sieber WJ, Browner D, Hoyt DB. Outcome after major trauma: 12-month and 18-month follow-up results from the Trauma Recovery Project. Journal of Trauma and Acute Care Surgery 1999;46(5):765-773.

In the methods section on page 4: September has only 30 days

- We thank the reviewer for his/her attention and changed this to 30^th^ of September.

**Comments reviewer 2**

The exclusion criteria includes patients over 80. This is a very common age group to sustain low energy pelvic/acetabular injuries. What were the reasons for exclusion of this particular group?

- Elderly pelvic fracture patients present an unique different patient cohort compared to their younger counterparts. We are aware of the fact that this elderly age group falls frequently with hip or pelvic fractures as a result. On the one hand it is difficult to investigate the functional QoL, such as the MAHS or MPS, due to a lack of mobility, on the other hand this age group suffers from many comorbid conditions. This makes it hard to differentiate the outcomes between pelvic related HRQoL and aging in general. We chose to perform a separate study of the age group with a pelvic fracture in the near future. A comparable sub-study which takes a close look at elderly hip fracture patients is already being performed as part of the BIOS study.

The impact of concomitant injuries and their impact upon their outcome scores could use more exploration. Whilst stratified for ISS there seems little discussion on the obvious effect that other injuries would have upon patient outcomes.

- We are aware of the fact that HRQoL is determined by more than sustaining a pelvic ring fracture. It is known that patients with pelvic ring fracture could suffer from associated injuries. Normally, the patient outcome is determined by all injuries. Therefore it could be important to investigate which part of the body can be correlated to a poor outcome.

In earlier versions of our manuscript we used multivariable mixed models to examine the course of HRQoL and functional outcome over time and between different Tile groups. Patient characteristics and injury-related characteristics (i.e. all 9 AIS regions, AIS severity of ≥ 2) were included in this analysis. Patients with pelvic ring fractures and associated injury of the upper extremity scored 0.10 lower on the EQ5D index (β= -0.10 (95% CI: -0.18 - -0.02)). Other associated injuries were not associated with a decreased HRQoL after a pelvic ring injury.

ISS is also known to be a risk factor for a decreased HRQoL. We chose to only include the ISS data (and not AIS data) in the multivariable mixed models because the subsets of AIS data included only small numbers of patients (table 1 patient characteristics). Adding too much prognostic factors influence and reduce the power of multivariable mixed models.

The use of the retrospective pre-injury score is fraught with some difficulties as noted by the authors. It is noted to be a prognostic factor for low post-injury scores. It is however unclear if this is a globally reduced score, or whether it is a reduced score relative to their baseline. It is unclear whether this is calculated relative to their pre-injury scores or whether this is calculated on the mean (we would not expect their pelvic injury to increase their QoL scores!)

- Figure 2 shows a global graphic of the mean EQ-5D VAS, Index and MPS at each time point post-injury. Patient-reported pre-injury EQ-5D index and VAS scores were 0.90 and 83 respectively. We also provided the average EQ-5D index summary score and VAS score for the general Dutch population, respectively 0.87 and 77.72. We now also added a small paragraph in the results section on the relationship between the pre-injury score and gender.

Table 4 shows the Beta’s from the multivariable mixed models investigating the predictors of reporting problems on the EQ-5D index and VAS. The pre-injury score was an important prognostic factor for a decreased HRQoL in both the EQ-5D VAS and index score. Indicating that baseline condition of the patient is an important prognostic factor for outcome after pelvic injury. The pre-injury score, as prognostic factor, was assessed over the first year after injury. Outcomes of EQ-5D index and VAS questionnaires 1 month, 3-, 6- and 12 months after trauma were compared to the outcome 1 week after trauma.

Is there any 24 month data? Its noted in the other BIOS article referenced ( Prevalence, recovery patterns and predictors of quality of life and costs after non-fatal injury: the Brabant Injury Outcome Surveillance (BIOS) study) That 24 month data points were collected. The authors have referenced 24 and 36 month outcomes from other papers, it would useful to include this data for comparison with the other studies referenced in the introduction.

- Thank you for this suggestions. Raw 24 months data is only collected of the EQ5D. However, due to lack of manpower, we have not yet got around to analyze this. This will hopefully be done in 2020-2021. We are looking forward to compare this data and combine it with the MPS and MAHS in the near future.

Figure 3, only demonstrates a single data point? Should there be separate points for both <65 and >65? Visually a line graph connecting the data points would be easier to follow than dots.

- We chose to visualize this figure using single data point because a “line” between the dots suggests there is data between these point. However, there is none. In case this is unclear for the editors or reviewers, we can modify this figure. We thank the reviewer for his/her accuracy. The MAHS was analyzed for the total population, and not specified into two age groups. We changed this in the manuscript.

As noted by the authors, pelvic fractures have a bimodality of injury presentation. Breakdown by high energy and low energy would be useful rather than purely fracture pattern type to highlight the two different disease patterns. Similarly this would give more meaning to the presence/absence of concomitant injuries and the types of shock on presentation, absence and presence of surgery. Furthermore, indications of requirements for other non-pelvic surgery related to their trauma would be useful, especially in the conservatively managed multi-trauma pelvic fracture patient.

- Our main outcome was HRQoL after a pelvic fracture. Tables 4, 5 and 6 show regression coefficients (95% CI) from the multivariable mixed models investigating the predictors of reporting problems on the EQ-5D index, VAS, MPS and MAHS, respectively. Gender, HET/LET, pelvic operation, ISS, age, education level and pre-injury score were all tested as prognostic factors of decreased HRQoL. Also, the different type of pelvic- and acetabular fractures were tested (Table 5 and 6 respectively).

The reviewer is suggesting to investigate pelvic fractures in terms of high- and low energy trauma. Table 4 is showing that this prognostic factor is not significant in the first year after trauma. Also the different type of pelvic- or acetabular fractures are not known as prognostic factors in the first year after trauma (table 5 and 6 respectively).

We agree with the reviewer that many articles focus on the different Tile or Judet-Letournel fracture types and that it could be of more clinical importance to focus on high or low energy trauma. However, for analyzing HRQoL in terms of high- or low energy trauma more patients are needed. Table 1 is showing the amount of LET/HET. Only a small amount of the patients had a high-energy presentation. The suggestions of the reviewer could be a good idea when performing an multicenter study with more patients.

**Supplemental table 1**

The HRQoL outcome over time and prognostic factors in the first year. Regression coefficients (95% CI) for EQ-5D and VAS for the first year after trauma assessed with univariable mixed models.

|  | **EQ-5D index score** | | | **EQ-5D VAS score** | | |
| --- | --- | --- | --- | --- | --- | --- |
|  | **Beta** | **95% CI** | **p-value** | **Beta** | **95% CI** | **p-value** |
| **Female** | -0.09 | -0.16 − -0.03 | <0.01 | -6.27 | -10.36 - -2.17 | <0.05 |
| **HET** | 0.00 | -0.07 – 0.07 | 0.98 | 0.87 | -3.38 – 5.11 | 0.69 |
| **Pelvic operation** | -0.04 | -0.11 – 0.04 | 0.33 | -1.28 | -5.83 – 3.28 | 0.58 |
| **Age ≥ 65 years** | 0.00 | -0.06 - 0.07 | 0.90 | 1.03 | -3.11 – 5.17 | 0.62 |
| **ISS 1-8*** | 0 |  |  | 0 |  |  |
| **ISS 9-15** | 0.03 | -0.07 – 0.12 | 0.56 | 2.17 | -3.91 – 8.25 | 0.48 |
| **ISS ≥ 16** | -0.10 | -0.18 - -0.02 | 0.02 | -7.02 | -12.04 - -2.00 | >0.01 |
| **Low education level*** | 0 |  |  | 0 |  |  |
| **Intermediate education level** | 0.03 | -0.04 – 0.10 | 0.43 | 0.67 | -4.07 – 5.40 | 0.78 |
| **High education level** | 0.04 | -0.05 – 0.13 | 0.35 | -0.66 | -6.25 – 4.93 | 0.92 |
| **Pre-injury score** | 0.62 | 0.44 – 0.79 | <0.001 | 0.39 | 0.27 – 0.51 | <0.001 |

*reference group.

Beta = regression coefficient, ISS = Injury Severity Score, CI = Confidence Interval, HET = High Energy Trauma

**Supplemental table 2**

The HRQoL outcome over time and prognostic factors in the first year analysed with univariable mixed models.

|  | **MPS < 65 years** | | | **MPS ≥ 65 years** | | |
| --- | --- | --- | --- | --- | --- | --- |
|  | **Beta** | **95% CI** | **p-value** | **Beta** | **95% CI** | **p-value** |
| **61A*** | 0 |  |  | 0 |  |  |
| **61B** | -3.03 | -10.88 – 4.81 | 0.44 | -0.13 | -10.56 – 21.67 | 0.49 |
| **61C** | -19.08 | -32.15 - -6.00 | <0.01 | 5.55 | -7.04 – 6.77 | 0.97 |
| **Female** | -7.79 | -15.53 - -0.05 | 0.05 | -7.65 | -13.53 - -1.76 | 0.01 |
| **HET** | 2.74 | -7.51 – 12.98 | 0.60 | 3.36 | -3.10 – 9.83 | 0.30 |
| **Pelvic operation** | -1.83 | -10.17 – 6.52 | 0.66 | -0.56 | -10.11 – 8.98 | 0.91 |
| **ISS 1-8*** | 0 |  |  | 0 |  |  |
| **ISS 9-15** | 7.20 | -3.71 – 18.12 | 0.19 | 3.25 | -4.76 – 11.26 | 0.42 |
| **ISS ≥ 16** | -4.89 | -13.36 – 3.58 | 0.25 | -9.28 | -19.44 – 0.89 | 0.07 |
| **Low education level *** | 0 |  |  | 0 |  |  |
| **Intermediate education level** | 0.79 | -8.67 – 10.25 | 0.87 | 3.65 | -3.39 – 10.69 | 0.30 |
| **High education level** | 7.72 | -3.31 – 18.75 | 0.17 | 6.21 | -2.05 – 14.48 | 0.14 |

*reference group.

Beta = regression coefficient, ISS = Injury Severity Score, CI = Confidence Interval, HET = High Energy Trauma

**Supplemental table 3**

The HRQoL outcome over time and prognostic factors in the first year analyzed with univariable mixed models.

|  | **Merle d’Aubigne hip score** | | |
| --- | --- | --- | --- |
|  | **Beta** | **95% CI** | **p-value** |
| **Elementary fracture** | 0* | - | - |
| **Associated fracture** | -1.38 | -2.49 - -0.27 | 0.02 |
| **Female** | -0.02 | -1.49 – 1.45 | 0.98 |
| **HET** | 1.36 | 0.15 – 2.56 | 0.03 |
| **Pelvic operation** | -0.47 | -1.64 – 0.69 | 0.42 |
| **Age ≥ 65 years** | -0.39 | -1.55 – 0.78 | 0.51 |
| **ISS 1-8*** | 0 | - | - |
| **ISS 9-15** | 0.13 | -1.95 – 2.22 | 0.90 |
| **ISS ≥ 16** | 0.11 | -1.50 – 1.73 | 0.89 |
| **Low education level *** | 0 | - | - |
| **Intermediate education level** | 0.36 | -1.04 – 1.76 | 0.21 |
| **High education level** | 0.97 | -0.55 – 2.50 | 0.61 |

*reference group.

Beta = regression coefficient, ISS = Injury Severity Score, CI = Confidence Interval, HET = High Energy Trauma
